# Chronic inflammation and quality of life in older adults: a cross-sectional study using biomarkers to predict emotional and relational outcomes

**DOI:** 10.1186/s12955-014-0141-0

**Published:** 2014-09-28

**Authors:** Alexandra CH Nowakowski

**Affiliations:** Department of Behavioral Sciences and Social Medicine, College of Medicine, Florida State University, 1115 West Call Street, Suite 3200, Tallahassee, FL 32306-4300 USA

**Keywords:** Inflammation, Quality of life, Health, NSHAP, Chronic conditions, Quantitative

## Abstract

**Background:**

This study explores relationships between chronic inflammation and quality of life, making a case for biopsychosocial modeling of these associations. It builds on research from social and clinical disciplines connecting chronic conditions, and inflammatory conditions specifically, to reduced quality of life.

**Methods:**

Data from Wave I of the National Social Life, Health, and Aging Project are modeled using ordinal logistic and ordinary least-squares regression techniques. Inflammation is measured using C-reactive protein; quality of life is conceptualized as happiness with life overall as well as intimate relationships specifically.

**Results:**

For most NSHAP participants, chronic inflammation significantly predicts lower odds of reporting high QoL on both emotional and relational measures. Social structural factors do not confound these associations. Inconsistent results for participants with very high (over 6 mg/L) CRP measurements suggest additional social influences.

**Conclusions:**

Findings echo strong theoretical justification for investigating relationships between CRP and QoL in greater detail. Further research should explore possible mediation of these associations by sociomedical sequelae of chronic disease as well as social relationship dynamics. Elaboration is also needed on the mechanisms by which social disadvantage may cause chronic inflammation.

## Background

Research on inflammation has become increasingly widespread throughout the sociomedical sciences in recent years. New datasets integrating biological data along with information about other types of health (emotional, behavioral, social, etc.) have allowed scholars to explore questions about how non-biological factors may predispose people to inflammation. Much of this work has focused on chronic inflammation, which may play a role in a wide variety of health conditions.

Likewise, researchers have long suspected that these conditions can shape social opportunities and relationships. Chronic conditions may thus impact quality of life (QoL) as people move through the experience of living with chronic disease. A robust literature on chronic disease and QoL exists in multiple sociomedical disciplines, including sociology. Yet comparatively little research exists on how inflammation itself impacts QoL. Even less research engages biomarker data—rather than diagnosis status or other clinical indicators—to capture underlying inflammatory pathology that may influence QoL.

This study contributes to a growing literature on relationships between inflammation and QoL. Specifically, it lays a foundation for sociological inquiry on inflammatory biomarkers as determinants of social and emotional health. It supports more nuanced analyses of inflammation’s influence on QoL in social context. Likewise, it brings together literatures from multiple fields to recommend a biopsychosocial understanding of how inflammation impacts social life and vice versa.

### Theoretical foundations

The inflammatory biomarker C-reactive protein (CRP) has a strong evidence basis for use in sociomedical research. Bodies produce CRP in response to inflammation [1-3]. CRP gets deposited wherever swelling occurs—including joints, mucous membranes, and muscles [3]. This substance is generated when inflammation of any type is present [1,2]. Consequently, it can capture inflammation from a wide variety of health conditions, even if the person has no diagnoses [3]. While CRP levels can reflect a certain amount of acute inflammation, they are particularly good for capturing chronicity.

Acute inflammation from injury or pathogenic illness is unlikely to introduce significant differential bias. Elevations in CRP levels from acute inflammation tend to be not only temporary, but also small [3]. By contrast, baseline CRP levels are generally stable over time [4]. Average CRP values increase with age, but the relative distribution of values according to health status remains largely stable over time [3,4]. Likewise, while baseline CRP levels cannot be used to diagnose any particular disease [5], high levels do provide excellent evidence of generalized inflammation. Elevated levels can also indicate progression of inflammation in people with chronic conditions [3,5].

High levels of CRP circulating freely in the blood generally indicate widespread inflammation affecting multiple tissues [4]. Indeed, researchers have discovered that levels of freely circulating CRP are determined exclusively by the body’s rate of synthesizing this substance [6]. Likewise, acute conditions are unlikely to elevate the synthesis rate substantially or for very long [4]. In fact, CRP levels often drop rapidly when synthesis stops [4]. CRP thus constitutes a good predictor of chronic inflammation for a variety of different disease populations, despite its inherent limitations.

Only a few published studies have explicitly focused on the relationship between CRP and QoL [7-9]. While these studies do not delve deeply into potential psychosocial dynamics of the association between CRP and QoL, they do find that elevated levels of CRP significantly predict diminished levels of QoL [7]. An excellent foundation thus exists for further work on CRP and QoL using generalized linear modeling techniques. Researchers in a variety of disciplines can now extend this foundation to more diverse outcomes that capture different elements of overall QoL. Scholars can also explore synergistic effects of biological and social mechanisms on QoL, and potentially unique dynamics of these phenomena in older adults. Given robust linkage in sociological literature between health status and self-rated QoL [10], a strong theoretical connection exists between CRP and QoL outcomes.

Although research on CRP specifically as a determinant of QoL remains largely absent from the literature, research on chronic inflammatory disease and its relationship to QoL has expanded greatly within the past 20 years. Several researchers have focused on general associations between specific inflammatory chronic conditions and health outcomes, finding significant associations between symptom severity and reductions in QoL [11,12]. Recent research also suggests dynamic relationships between inflammation and patient affect as a potential mediator of associations between chronic inflammation and QoL for a variety of health conditions [13-15].

Literature on specific inflammatory diseases largely mirrors these general findings concerning inflammatory disease and QoL. For example, literature on arthritis documents robust linkage between severity of inflammation and reductions in QoL, both acutely and over time [16-24]. This literature also includes research on: asthma [25]; interstitial cystitis [26-36]; irritable bowel syndrome [37]; inflammatory bowel disease [37]; chronic ulcerative colitis [38]; Crohn’s disease [39,40]; non-specific bowel inflammation [41-44]; chronic prostatitis [45]; vulvuodynia [46]; and non-specific genital inflammation [46]. These scholars have focused on a wide range of QoL outcomes, spanning from self-rated enjoyment of life to sexual satisfaction to economic livelihood.

Psychosocial mechanisms implicated in the relationship between chronic inflammation and reduced QoL include: adverse interactional dynamics [47,48]; internal stress processes [49,50]; emotional strain of coping with negative social consequences of illness [13,51]; physical, emotional, and sexual abuse [47,48]; depression [48]; sexual dysfunction [36]; pursuit of “health-related normality” [52]; fear of having to deal with their symptoms in public places or in visible ways [43,51]; feelings of isolation and alienation [53,54]; lack of social support from others [55]; frustration in seeking a diagnosis [50,56]. Comorbidities are common among people with chronic inflammation [57], and often exacerbate the negative consequences of inflammation for QoL [35].

Both the literature on generalized inflammation and the literatures on specific inflammatory diseases as predictors of QoL indicate that inflammation can cause changes in QoL. Extant research also suggests that stress can cause and/or exacerbate inflammation itself [49,50]. Consequently, people whose QoL is already low for reasons not related to inflammatory pathology may experience higher levels of inflammation if their life circumstances induce psychological distress. Overall extant literature indicates that inflammation can cause reductions in QoL. These reductions may in turn be exacerbated by psychological distress [8] and resultant increases in allostatic load [58], creating a vicious cycle of cumulative inequality in health over time [59].

As suggested previously, diagnosis remains highly problematic because many people with these diseases go untreated, never receive an accurate diagnosis, or find that no clinically recognized diagnosis yet exists for their condition [50,56]. Likewise, many patients who do receive one diagnosis do not ever learn the full extent of their condition due to the siloized structure of specialty medicine in the United States. These issues may affect women disproportionately, compounding an already significant array of disadvantages in health [60,61]. These studies thus present a strong case for studying associations between CRP and QoL in lieu of using diagnosis information.

In studying these associations, scholars should note that chronic inflammation itself may reflect the influence of multiple social factors. This is especially true for older adults [62], as cumulation processes may manifest strongly [59]. Link and Phelan’s [63] review of advancements in fundamental causation theories supports both this argument and the idea that social factors may condition the impacts of inflammation on QoL once present [63]. The authors illuminate the potential for both material and psychosocial resources to impact people’s ability to cope with disease.

Research on resource multiplication illustrates the potential for relatively small disadvantages in psychosocial resiliency to compound into major consequences for health and QoL. This reasoning again introduces a cumulation perspective that mirrors the “weathering” phenomenon observed in Geronimus and colleagues’ [58,60,61] work on physical health. Consequently, data on older adults may offer particular insight into the effects of chronic inflammation on QoL across the life course. Multiple research teams [62,64] have conducted meaningful inquiry into chronic inflammation among older adults using the National Social Life, Health, and Aging Project (NSHAP) dataset collected by Linda Waite and colleagues.

## Methods

### Data and subject selection

This study uses data from Wave I of the NSHAP. This biopsychosocial dataset provides information on physical, mental, and social health among United States residents aged 57 to 85. Consequently, the NSHAP is an excellent resource for interdisciplinary scholars of health and QoL in older adults [65]. This dataset allows researchers to explore the synergistic relationships between biological, psychological, and social factors that may influence health status and perceived quality of life.

The NSHAP dataset was developed between 2005 and 2006 by researchers at the University of Chicago. As such, the NSHAP includes data on 3,005 individuals living in and around Chicago, Illinois. Oversampling of specific demographic groups (African Americans, Latinos, males, and persons 75 to 85 years of age) allows the dataset to be nationally representative [66]. Data for the NSHAP are collected via a combination of in-home interviews, supplemental questionnaires, blood spot blotters, and saliva cultures. As of Summer 2014 the NSHAP remains essentially cross-sectional, with only two waves of data available.

Life course sociology literature increasingly suggests that disadvantage in psychosocial outcomes accumulates over time from adverse social experience [67-73]. While the NSHAP data remain largely cross-sectional at present, the inclusion criteria guarantee at least 57 years elapsed between birth and data collection for a given subject. Analyzing an older population cross-sectionally allows researchers to capture long-term impacts from biosocial exposures that began in early life [67,68]. Consequently, the NSHAP can illuminate potential cumulation of disadvantage in health and QoL from chronic inflammation due to inherited conditions.

QoL was examined in two different ways: emotionally (via self-reports of general happiness) and relationally (via self-reports of overall happiness with intimate relationships). Extant research suggests that relevant biological and social predictors may not influence all domains of QoL equally [74]. In addition, factor analysis for the two QoL outcomes yielded relatively high values for uniqueness, suggesting that discrete information about QoL was captured by each of the two measures. Assessing different types of QoL thus facilitated broader inference about the potential impact of chronic inflammation on these outcomes, and the social factors that may contribute to these patterns.

The main predictor for both QoL outcomes was CRP. This biomarker has a strong evidence basis supporting its use in biosocial research on inflammation. CRP also provides the best evidence of chronic inflammation among available variables in the NSHAP. In assessing the potential influence of CRP on QoL, the possible role of numerous social covariates was also examined as outlined in Table 1. These covariates were considered as both potential confounders of relationships between inflammation and QoL, and fundamental causes of inflammation itself.

**Table 1:** **Descriptions and summary statistics for included variables**

**Construct**	**Variable**	**Units**	**Cases**	**Mean**	**St. Dev.**	**Min.**	**Max.**
Quality of life	Generally feeling happy	Points	2,995	3.58	0.88	1	5
	Happiness with relationships		2,890	5.89	1.61	1	7
Chronic inflammation	C-reactive protein	mg/L	1,939	3.20	6.03	0	100
Sex	Female	Yes/No	3,005	0.52	0.50	0	1
	Male			0.48	0.50		
Age	Years of age	Years	3,005	69.3	7.85	57	85
	57 to 64	Yes/No	3,005	0.34	0.47	0	1
	65 to 74			0.36	0.48		
	75 to 85			0.30	0.46		
Education	Years of education	Years	2,971	12.48	4.10	0	32
	Less than high school	Yes/No	3,005	0.23	0.42	0	1
	High school or equivalent			0.26	0.44		
	Some college			0.28	0.45		
	Finished college			0.22	0.41		
Race	White	Yes/No	2,993	0.70	0.46	0	1
	Any minority			0.30	0.46		
	Black			0.17	0.38		
	Hispanic			0.10	0.30		
	Other race			0.02	0.15		
Income	Household earnings	$/year	2,124	51,264	76,559	0	1,800,000
Social support and communication	Open with partner	Points	2,012	2.71	0.54	1	3
	Open with friends		2,704	2.02	0.73		
	Relying on family	Points	2,793	2.57	0.65	1	3
	Relying on friends		2,680	2.30	0.71		
Relationship status	Married	Yes/No	3,005	0.60	0.49	0	1
	Cohabitating			0.02	0.14		
	Separated			0.02	0.13		
	Divorced			0.11	0.31		
	Widowed			0.22	0.41		
	Never married			0.04	0.19		

Using correlational tables, all variables were checked for unique value as covariates—i.e., low collinearity. Certain covariates (e.g., feeling able to communicate openly with intimate partners) also correlated somewhat with one or both outcome variables. However, this did not present a methodological problem for two reasons. First, the NSHAP data documentation explains that the outcome variable measuring happiness with intimate relationships captures a variety of factors that may influence this perception, including emotional satisfaction, physical satisfaction, quality of communication, feelings of being able to rely on another person, and a variety of other elements [66]. Second, one of the goals of this study was to generate hypotheses about potential social mediators of observed associations between CRP and QoL.

Correlational tables were also used to check for differential attrition owing to the lack of successful CRP measurements for about 35% of NSHAP participants. Challenges with inference related to the original measurement strategies in the NSHAP were also noted. For example, the dataset captures information on sex only as a binary construct. It also does not (despite calling its sex variable “gender”) collect any information on gender identity. Assessing possible effects from ethnoracial background presented similar challenges. Despite the NSHAP’s nationally representative sampling strategy, data could only be used for participants whose CRP serolevels had been successfully measured. Analyses related to race thus focused primarily on the potential impact of identifying as a member of any racial minority group.

As suggested above, some variables in the NSHAP may have problematic values due to measurement error. This concern is most pressing for the CRP variable. Although most values are clustered within a range of 10 mg/L, some values are closer to 50 and one person appears to have a value of 100. Comparing the CRP variable’s median value (1.531 mg/L) to its mean value (3.19 mg/L) suggests that outliers may be a problem for this measure. Since CRP levels usually remain in the single digits even for people with severe inflammation, these very high values are more likely attributable to measurement issues. Indeed, data documentation for the NSHAP suggests that most implausible measurements on CRP owe to issues with data collection and processing.

This study was approved by the Florida State University Human Subjects Committee. All data management and analysis was conducted using Stata Version 12.

### Analytic techniques

Net associations between chronic inflammation and QoL were examined. Hypotheses were tested concerning (1) potential unique effects of inflammatory pathology on QoL, and (2) the ability of CRP to mediate associations between fundamental social determinants and QoL. Recommendations were developed for further investigation of these hypotheses in future studies.

Generalized linear modeling techniques, specifically ordinal logistic regression, were used to regress QoL outcomes on CRP. While both QoL variables are numeric and additive in nature, they do not approximate continuity because of the relatively limited numbers of response categories (between 5 and 7) available for each item. Furthermore, QoL data in the NSHAP have significant left skew, consistent with evidence from the literature that life satisfaction is often relatively high among older adults [75].

Consequently, either ordinal logistic (ologit) regression or ordinal probabilistic (oprobit) regression was indicated [74]. Sensitivity analyses using both frameworks were performed to ensure that results did not differ substantially; ologit models are presented for ease of interpretation. Models with and without social structure and relationship covariates (enumerated in Table 1) were generated to check for potential confounding influences. Pursuant to the above discussion of parametric versus non-parametric methods, OLS regression was used to conduct additional sensitivity analyses, but did not meaningfully alter findings. Results from non-parametric methods are thus presented for optimal substantive validity.

In exploring the potential influence of social structure and relationship factors, OLS regressions were generated of CRP on social structure and relationship predictors respectively. These models built on findings from previous research [62,64] suggesting that social factors could fundamentally cause chronic inflammation. Both the OLS models and the more complex ologit models allowed for the development of mediation frameworks for formal analysis in future studies.

Model diagnostics were performed to check regression assumptions and assess the influence of possible outlying data. For ologit models, these included likelihood ratio and Wald tests of the proportional odds/parallel regression assumption. For OLS models, these included Breusch-Pagan tests for heteroskedasticity, and re-estimation of models using Huber-White standard errors when indicated. Potential outliers were investigated by generating standardized residuals with the “predict” command and plotting them by case identification number. When indicated, models were re-estimated excluding observations with standardized residual values greater than 2. While this process often strengthened findings slightly, unadjusted findings are presented to maintain conservatism in interpreting results.

Additional sensitivity analyses were conducted to address potential problems related to sampling and measurement. Alternate models were computed using Stata’s “survey” and/or “cluster” prefixes to incorporate sampling weights. The “robust” omnibus corrector option was also used to adjust for a variety of methodological issues. However, neither of these approaches significantly impacted substantive findings. This likely owed to the size and diversity of analytic samples, as well the active incorporation of constructs addressed by NSHAP sampling weights. Unadjusted results are thus presented for ease of interpretation.

To compare models with and without statistical controls, fit statistics were computed, including pseudo R-squared values and information criteria. This helped to generate hypotheses about whether or not inflammatory pathology can confer additional effects on QoL in excess of the social factors that can fundamentally cause chronic inflammation. Findings from this study recommend exploring chronic inflammation in two distinct contexts: (1) as a fundamental biological cause of QoL, and (2) as a mediator in relationships between fundamental social determinants and QoL outcomes.

## Results

### Summary of findings

Ordinal logistic regression models of the two QoL outcomes on the CRP predictor were generated and examined. Simply regressing QoL scores on the raw CRP variable did not prove useful because this general level of analysis masked more nuanced trends in the data. Several sets of bivariate models were thus created, using different methods of coding the CRP variable, using different sets of inclusion criteria for CRP and income, and using models stratified by different levels of serum CRP. Rather than attempting to select a single series of final models, general trends were noted across different inclusion criteria and levels of inflammation. This manuscript takes a conservative approach to presenting findings. All values reported come from regressions using the full sample for each outcome variable, with no additional inclusion criteria.

Highly significant negative associations were identified between CRP levels and both QoL variables. Specifically, CRP significantly predicted NSHAP participants’ odds of reporting high levels of general happiness and relationship happiness. Net associations with CRP for these QoL measures mirrored one another in terms of magnitude. When exponentiated, both raw coefficients for the CRP variable yielded odds ratios of 0.90 (p < 0.001) for general happiness and 0.92 (p < 0.01) for happiness with relationships. Among people in the NSHAP sample, each additional milligram per liter of serum CRP is thus associated with 8 to 10 percent lower odds of experiencing high levels of QoL when measured either emotionally or relationally.

However, these findings only held true for levels of CRP below 6 mg/L. These distinctions were explored using regressions stratified by a 6 mg/L CRP threshold. Within each group, CRP was preserved as a continuous independent variable and used to predict QoL. People with more than 6 mg/L of serum CRP did not exhibit a significant association between their levels of the inflammatory biomarker and QoL on using either outcome measure. Differences in sample sizes may partially account for this dichotomy: Samples for the CRP < 6 mg/L group were 1,683 and 1,626 for emotional and relational QoL outcomes respectively, versus only 255 and 246 for the CRP > 6 mg/L group. The discussion section of this manuscript explores potential (beyond simple sampling power issues) for which significant relationships were not observed between CRP and QoL above 6 mg/L.

Ensuing analyses thus focus only on results for people with less than 6 mg/L of serum CRP. Sample sizes for each stratum of inflammatory biomarker values are also displayed. For each outcome, the group of NSHAP participants with CRP levels below 6 mg/L represented approximately 87 percent of total variation in inflammatory biomarker values contained within the full dataset. Indeed, a growing body of clinical literature on CRP indicates that stratifying out values in the upper single digits and beyond can generally capture the vast majority of true variation within a given study population [62]. Even among older adults, pronounced inflammatory pathology often appears at very small numeric values of CRP [62]. Hypotheses were thus generated about how differences in inflammatory biomarker levels may become important sources of social inequality.

**Figure 1:**
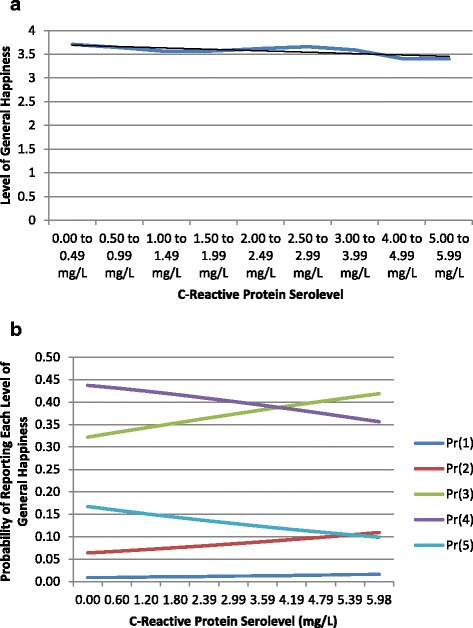
**General happiness predicted by inflammatory biomarker levels. (a)** Distribution of Emotional QoL Scores by CRP Serolevel. **(b)** Probability of Emotional QoL Scores by CRP Serolevel.

**Figure 2:**
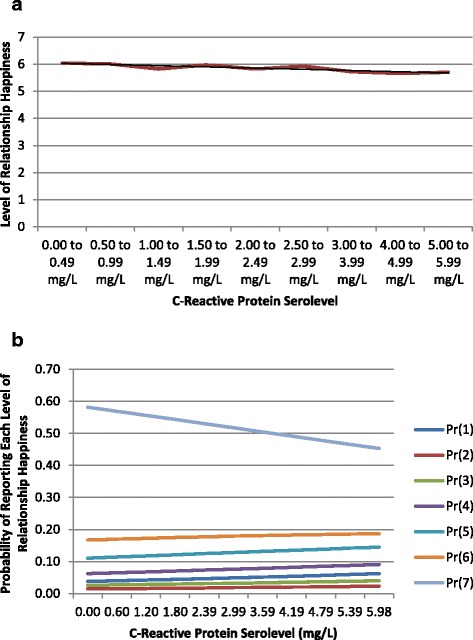
**Relationship happiness predicted by inflammatory biomarker levels. (a)** Distribution of Relational QoL Scores by CRP Serolevel. **(b)** Probability of Relational QoL Scores by CRP Serolevel.

Figures 1a through 2b illustrate graphically how the probability of reporting different levels of QoL on each relevant measure varies according to CRP readings. Figures 1a and 2a show how scores for each outcome vary according to levels of CRP. By contrast, Figures 1b and 2b show variation within specific levels of the outcome variable across different amounts of CRP. Taken together, these figures illuminate both overall trends and specific dynamics of how chronic inflammation may impact emotional and relational QoL.

Figures 1a and 2a illustrate that while significant relationships between CRP serolevels and QoL can be observed for both the emotional and relational measures of QoL. Figures 1b and 2b add detail by illustrating two complementary patterns. Higher levels of CRP generally predict lower odds of reporting high levels of QoL on each outcome measure. By contrast, lower levels of CRP predict higher odds of reporting higher levels of QoL.

These graphics also reveal nuances of the observed relationships between inflammatory biomarker levels and QoL. Specifically, while clear overall correlations (shown with trendlines) present themselves between CRP and both QoL outcomes, the curves of best fit for these data do not closely approximate linearity in either case. Sociomedical phenomena that may contribute to these patterns are explored further in the discussion section.

### Alternate model specifications

Bivariate models for both outcomes were also re-estimated incorporating social structural factors. These covariates represented formal statistical “controls” that could help to illuminate any potential confounding from structural disadvantage. Likewise, significant but non-confounding results for social structure covariates could illustrate potential fundamental causation of chronic inflammation by different types of disadvantage in social status and position.

Re-estimating models for both outcomes using social structure and relationship covariates did indeed return significant results for a variety of social factors. With respect to social structure, both female sex (β = 0.288, p < 0.001) and racial minority identity (β = 0.282, p < 0.01) predicted higher levels of CRP. By contrast, each additional 10,000 dollars of annual income (β = −0.001, p < 0.05) predicted slightly lower levels of CRP. Additional years of age did not significantly predict differences in CRP serolevels. With respect to social relationships, being married (β = −0.413, p < 0.01) and feeling able to communicate openly with intimate partners (β = −0.213, p < 0.05) both predicted lower levels of CRP. Other social relationship factors such as feeling able to communicate openly with friends, feeling able to rely on family members, and feeling able to rely on friends did not significantly predict differences in CRP serolevels.

Yet these results did not generally indicate a confounding influence by any of the structural factors assessed. In fact, some social factors (including race for the emotional outcome and income for the relational one) became non-significant when incorporated into more complex models for each QoL outcome. In general, results from computing more complex models suggested that fundamental causation of inflammation by structural disadvantage was occurring, in addition to potential independent influences on QoL from these same factors. Preliminary analyses of fundamental causation corroborated work by other scholars [62,64] suggesting that disadvantage in a variety of social domains can fundamentally cause chronic inflammation.

A notable exception to the overall pattern of CRP retaining at least some predictive significance was the relational QoL outcome when modeled incorporating social relationship factors. Upon incorporating these covariates, the CRP term became non-significant. However, the literature suggested that because social relationship dynamics can change dramatically in response to incipent or increasingly severe chronic disease [54,55] these factors were more likely to mediate associations between chronic inflammation and QoL rather than confounding them. Similar patterns appeared with the emotional QoL outcome, for which CRP remained a significant (albeit less so) predictor in the full model. Given that QoL outcomes directly addressing social relationships are likely to respond more strongly to changes in these dynamics, more sophisticated mediation analyses using longitudinal data should be conducted in the future.

Models were also re-estimated using interaction terms to check for moderating influences by social structure factors that showed independently significant relationships with one or both QoL outcomes. None of these interaction terms showed any statistical significance when incorporated into more complex models, and are thus not presented in depth using tables or graphs. These null results do not constitute unilateral evidence that structural disadvantage cannot moderate associations between chronic inflammation and QoL. Rather, they suggest that key aspects of social structure likely exert their main influences farther “upstream” [76] than stages of causation captured by moderation frameworks. Indeed, as with the analyses incorporating statistical controls as discussed above, preliminary moderation analyses merely provided more support for the idea that social factors can fundamentally cause chronic inflammation—and perhaps also QoL as a partial function of this inflammatory pathology.

## Discussion

### Implications of key findings

Findings from this study suggest that the progression of chronic inflammatory disease may exert a significant negative impact on QoL in both emotional and social domains. This study establishes a conceptual foundation that demonstrates the feasibility and importance of conducting more detailed and sophisticated research on the formative and summative sociomedical dynamics of chronic inflammation. Likewise, it can help to corroborate previous research on the fundamental social causes of inflammation itself, especially findings from other studies using NSHAP data [62].

While preserving a robust and representative sample of people in the NSHAP, stratifying my analyses by CRP levels below and above 6 mg/L also limited the extent to which outlying values attributable to measurement error could skew findings. Using evidence-based stratification techniques optimized internal validity without dismissing the potential contributions of very high CRP values to the overall study results. This facilitated consideration of why the much smaller samples of people with biomarker levels greater than 6 mg/L showed no significant associations between chronic inflammation and QoL, whereas their peers had markedly different results. As noted previously, among people with fewer than 6 mg/L of the CRP marker, chronic inflammation was significantly associated with both emotional and relational QoL.

Other sociological studies of QoL reflect that different QoL outcomes may be more or less salient to different people. They also suggest that depending on the particular elements of QoL they capture, these same measures may be more or less sensitive to influence from specific biological and social forces. When measured using CRP, chronic inflammation appears to have the potential to influence people’s overall happiness, as well as their satisfaction in intimate relationships, to a small but consistent degree. Other QoL outcomes may show different patterns of association with CRP.

This same literature has important implications for the remainder of my analyses of possible relationships between CRP and QoL. Any associations documented in older populations are likely to be more pronounced among younger groups, as older individuals may expect a certain amount of decline in health and functionality as a normal part of the experience of growing older. The social role of “older adult” in contemporary American society thus includes changes in physical health and functionality, despite growing pressure to minimize these changes as much as possible. Demonstrating evidence of impairment in the course of performing their prescribed social role thus affords older adults a certain amount of legitimacy and credibility, even if they experience very large declines in health and/or functionality.

Likewise, presenting evidence of diminished health and/or functionality can lead older adults to begin receiving assistance, either informally from family members or formally from home health professionals. Extant sociological literature suggests that while people often experience negative feelings related to loss of independence when entering into a caregiving arrangement, receiving care from other people can also exert significant positive impacts on QoL. Researchers in a variety of disciplines have documented how living with any type of persistent illness can produce feelings of isolation and loneliness, both of which negatively affect QoL. These feelings can become particularly pronounced for people who experience a level of functional disability substantial enough to make living alone difficult, but not severe enough to warrant seeking outside assistance. Taken together, these trends may explain why a significant association between CRP levels and QoL outcomes did not emerge for NSHAP participants with CRP levels in excess of 6 mg/L.

As noted in the results section, visual analysis of NSHAP data revealed nuances in the relationships between specific levels of CRP and the odds of reporting high QoL across both outcome measures. While overall correlations between CRP and QoL were consistently negative and clear trendlines emerged, the curves of best fit for these data did not entirely resemble straight lines. This may owe in part to measurement considerations, especially the fact that while higher levels of CRP do predict chronic inflammation in an overall population, specific individuals may have levels significantly higher than average without experiencing significant pathology.

The higher average levels of CRP found among female-bodied NSHAP participants may speak to this phenomenon. As noted in the literature review, females tend to have higher average levels of inflammatory biomarkers regardless of disease status when compared to their male peers. However, newer research suggests that higher levels of CRP in females may owe strongly to underlying social inequalities that impact females disproportionately [61].

Differences in average levels of CRP among subgroups within the full NSHAP sample may also stem from sociomedical phenomena related to the lived experience of chronicity at particular levels of inflammatory pathology. Across different levels of disease pathology, people’s expectations for their health and resultant QoL may differ. Extant literature shows strong associations between cognitive dissonance—the mental anguish people experience when reality differs unfavorably from expectations—and reduced QoL among people who develop chronic conditions, including inflammatory diseases.

The fact that social structure factors did not confound observed relationships between CRP levels and either QoL outcome indicates that these forces may exert other types of influences. Fundamental causation [77] of chronic inflammation by structural disadvantage is one of the most likely ways in which these influences may operate. Indeed, previous research using the NSHAP [62] supports conceptualizing chronic inflammation as a socially conditioned outcome. Likewise, a variety of other studies have shown strong associations between structural disadvantage and the onset of different chronic inflammatory conditions [59,78,79]. Future research should thus analyze inflammatory biomarkers themselves as potential mediators of relationships between social disadvantage and QoL.

Strengths of this study include background and perspectives from multiple disciplines, parsimonious model specifications, and use of data on older adults. The interdisciplinary approach used in this research helps to bridge gaps in prior work on inflammation, health, social life, and QoL. As such inquiry into chronic inflammation’s potential effects on QoL remains relatively new, parsimonious models were used to guard against reporting of significant associations in cases where none were present. The choice of NSHAP as a data source, and specifically the advanced ages of its participants, allowed for insight into potential cumulation of disadvantage in QoL. This advantage persisted despite the limited number of waves currently available in the NSHAP.

Limitations of this study include lack of longitudinal data, challenges with data capture in the NSHAP, and narrow generalizability. Two specific factors limiting generalizability are the advanced ages of participants and relatively high average levels of QoL among these individuals. Only two waves of NSHAP data are currently available, so the potential for using panel designs to study these phenomena remains limited. NSHAP researchers also experienced problems with certain measurements in the dataset, including the CRP assay. Although this study accounts for potentially problematic CRP values via stratification, it is nonetheless possible that some values in the focal range were also erroneous. Using data on older adults confers drawbacks in addition to the advantages noted above. Specifically, the relatively small range of ages captured by the NSHAP restricts the generalizability of these findings to late midlife and elderly adulthood. Similar processes may occur in children, adolescents, and younger adults without manifesting in the same ways.

Irrespective of the specific strengths and limitations of a particular study, it should also be noted that no research can unilaterally confirm or refute the potential influence of chronic inflammation on QoL. Rather, the statistical significance and reliability of results from this study lend support to the idea of inflammatory biomarkers as meaningful correlates of QoL. These findings likewise recommend further investigation of the topic in both community and clinical settings.

In addition to their consistent statistical significance, these findings may indeed possess some clinical significance. Specifically, the potential for a 1 mg/L difference in CRP levels to produce 8 to 10 percent lower odds of reporting high levels of QoL has important implications for people’s everyday lives. A person with severe inflammatory disease is likely to experience substantially elevated levels of CRP within a certain range [62]. Stratifying regression analyses using a cutoff value of 6 mg/L of CRP allowed for examination of variability across the vast majority of the analytic sample. For each computed model, the size of the population group with CRP serolevels between 0 and 6 mg/L exceeded that of its counterpart with levels in excess of 6 mg/L by approximately a factor of 8.

## Conclusions

Taken together, findings from analyses of CRP’s potential net effects on measures of happiness and relationship satisfaction suggest that chronic inflammation may be able to exert a significant negative impact on QoL for older adults. The findings themselves do not constitute firm evidence of a causal association between CRP levels and QoL. However, the robust associations documented between CRP and QoL across a variety of measures indicates that the relationship may be causal, and deserving of further exploration.

Scholars of aging and health should note that the above patterns hold true only for community-dwelling older adults—i.e., the specific population sampled by the NSHAP. Elderly persons residing in long-term care (LTC) settings are often discredited and ignored [80]. This can occur even for residents with extremely strong cognitive and/or physical functioning [80]. Indeed, persons who would have been eligible for participation in NSHAP but moved to long-term care facilities before the study began were censored from the sample. The potential influence of chronic inflammation on the social lives and QoL of older adults in LTC settings thus represents a priority for future research using other data.

Future research should also contextualize chronic inflammation within broader biopsychosocial models of QoL. Extant research suggests that social factors can act as fundamental causes [77] of both chronic inflammation [62,64] and QoL [75]. Specifically, disadvantage in one or more social domains predicts both increased inflammatory biomarker levels [62,64] and reduced QoL [75]. However, research is needed on whether or not chronic inflammation can mediate associations between structural disadvantage and QoL.

Likewise, associations between inflammatory biomarker levels and QoL may themselves be mediated by additional social factors. These potential mediating influences should be explored in future studies. Extant literature provides ample support for social interaction dynamics as potential mediators of associations between health status characteristics and QoL outcomes. For example, trouble in communicating with intimate partners can intensify the impact of disadvantage in health on QoL [81,82]. Loss of social support from friends and family members who become emotionally exhausted may also lead to sharper reductions in QoL among those with persistent inflammation [83].

Similar interactional factors may also moderate associations between inflammation and QoL. If the dynamics of people’s social relationships remain relatively unchanged by severity of inflammation, then associations between inflammatory biomarkers and QoL outcomes may vary according to initial levels of interactional advantage. For example, people who communicate openly with their intimate partners may find that dealing with challenges in health actually brings them closer [16,17]. In turn, social support received from intimate partners may help to protect health [84] and preserve QoL [85]. Conversely, people in strained relationships may find that their partners become distant and unsupportive as their health declines [54,83].

Finally, further research can benefit from incorporating longitudinal data, as well as more sophisticated regression frameworks such as Structural Equation Modeling (SEM). Though only two waves of NSHAP data are available currently, future research can leverage panel analysis techniques to illuminate potential mediation, moderation, and fundamental causation influences in greater depth. Likewise, techniques such as SEM can more accurately capture the simultaneous, synergistic impacts of multiple social and clinical factors. As more waves of NSHAP data become available, researchers can draw on these frameworks to achieve more nuanced and comprehensive understanding of how chronic inflammation may shape QoL in biopsychosocial context.

## Abbreviations

CRP: C-reactive protein
NSHAP: National social life, health, and aging project
OLS: Ordinary least-squares
QoL: Quality of life
SEM: Structural equation modeling

## References

[1] Shine B, De Beer FC, Pepys MB (1981). Solid phase radioimmunoassays for human C-reactive protein. Clin Chim Acta.

[2] Pepys MB, Baltz ML (1982). Acute phase proteins with special reference to C-reactive protein and related proteins (pentaxins) and serum amyloid A protein. Adv Immunol.

[3] Pepys MB, Hirschfield GM (2003). C-reactive protein: a critical update. J Clin Invest.

[4] Hutchinson WL, Koenig W, Fröhlich M, Sund M, Lowe GD, Pepys MB (2003). Immunoradiometric assay of circulating C-reactive protein: age-related values in the adult general population. Clin Chem.

[5] Szalai AJ, McCrory MA, Cooper GS, Wu J, Kimberly RP (2002). Association between baseline levels of C-reactive protein (CRP) and a dinucleotide repeat polymorphism in the intron of the CRP gene. Genes Immun.

[6] Vigushin DM, Pepys MB, Hawkins PN (1993). Metabolic and scintigraphic studies of radioiodinated human C-reactive protein in health and disease. J Clin Invest.

[7] Hamer M, Chida Y (2011). Life satisfaction and inflammatory biomarkers: the 2008 Scottish Health Survey. Jpn Psychol Res.

[8] Cummings DM, King DE, Mainous AG (2003). C-reactive protein, antiinflammatory drugs, and quality of life in diabetes. Ann Pharmacotherapy.

[9] Long H, Luo H, Chen P, Li Y (2011). Correlation among the levels of C-reactive protein and interleukin-18, quality of life, and lung function in patients with chronic obstructive pulmonary disease. PubMed abstract only—original article is in the Chinese-language journal. Zhong Nan Da Xue Xue Bao Yi Xue Ban.

[10] Mirowsky J, Ross CE (2003). Social Causes of Psychological Distress.

[11] Testa MA, Simonson DC (1996). Assessment of quality-of-life outcomes. N Engl J Med.

[12] Sprangers MA, de Regt EB, Andries F, van Agt HM, Bijl RV, de Boer JB, de Haes HC (2000). Which chronic conditions are associated with better or poorer quality of life?. J Clin Epidemiol.

[13] Epel ES, Blackburn EH, Lin J, Dhabhar FS, Adler NE, Morrow JD, Cawthon RM, Verma IM (2004). Accelerated Telomere Shortening in Response to Life Stress. Proceedings of the National Academy of Sciences of the United States of America: 7 December 2004; Washington DC.

[14] Finch CE, Crimmins EM (2004). Inflammatory exposure and historical changes in human life-spans. Science.

[15] Willson AE, Shuey KM, Elder GH (2007). Cumulative advantage processes as mechanisms of inequality in life course health. Am J Sociol.

[16] Manne SL, Zautra AJ (1989). Spouse criticism and support: their association with coping and psychological adjustment among women with rheumatoid arthritis. J Pers Soc Psychol.

[17] Manne SL, Zautra AJ (1990). Couples coping with chronic illness: women with rheumatoid arthritis and their healthy husbands. J Behav Med.

[18] Whalley D, McKenna SP, De Jong Z, Van der Heijde D (1997). Quality of life in rheumatoid arthritis. Ballieres Clin Rheumatol.

[19] Kosinski M, Kujawski SC, Martin R, Wanke LA, Buatti MC, Ware JE, Perfetto EM (2002). Health-related quality of life in early rheumatoid arthritis: impact of disease and treatment response. Am J Man Care.

[20] Sokoll KB, Helliwell PS (2001). Comparison of disability and quality of life in rheumatoid and psoriatic arthritis. J Rheumatol.

[21] Bell MJ, Bombardier C, Tugwell P (1990). Measurement of functional status, quality of life, and utility in rheumatoid arthritis. Arthritis Rheum.

[22] Foster HE, Marshall N, Myers A, Dunkley P, Griffiths ID (2003). Outcome in adults with juvenile idiopathic arthritis: a quality of life study. Arthritis Rheum.

[23] Picavet HSJ, Hoeymans N (2004). Health related quality of life in multiple musculoskeletal diseases: SF-36 and EQ-5D in the DMC3 study. Ann Rheum Dis.

[24] Gladman DD, Antoni C, Mease P, Clegg DO, Nash P (2005). Psoriatic arthritis: epidemiology, clinical features, course, and outcome. Ann Rheum Dis.

[25] Schmaling KB, Afari N, Hops H, Barnhart S, Buchwald D (2009). Change in airflow among patients with asthma discussing relationship problems with their partners. J Health Psychol.

[26] Held PJ, Hanno PM, Wein AJ, Pauly MV, Cahn MA, Hanno PM (1990). Epidemiology of Interstitial Cystitis: 2. Interstitial Cystitis.

[27] Ratner V (2001). Interstitial cystitis: a chronic inflammatory bladder condition. World J Urol.

[28] Moldwin RM, Sant GR (2002). Interstitial cystitis: a pathophysiology and treatment update. Clin Obstet Gynecol.

[29] Nickel JC (2004). Interstitial cystitis: a chronic pelvic pain syndrome. Med Clin North Am.

[30] Rosamilia A (2005). Painful bladder syndrome/interstitial cystitis. Best practice & research. Clin Obstet Gynecol.

[31] Hanno PM, Walsh PC, Retik AB, Vaughan ED (2002). Interstitial Cystitis and Related Disorders. Campbell’s Urology.

[32] Van De Merwe JP, Yamada T, Sakamoto Y (2003). Systemic aspects of interstitial cystitis, immunology and linkage with autoimmune disorders. Int J Urol.

[33] Rothrock NE, Lutgendorf SK, Hoffman A, Kreder KJ (2002). Depressive symptoms and quality of life in patients with interstitial cystitis. J Urol.

[34] Rothrock NE, Lutgendorf SK, Kreder KJ (2003). Coping strategies in patients with interstitial cystitis: relationships with quality of life and depression. J Urol.

[35] Novi JM, Jeronis S, Srinivas S, Srinivasan R, Morgan MA, Arya LA (2005). Risk of irritable bowel syndrome and depression in women with interstitial cystitis: a case–control study. J Urol.

[36] Nickel JC, Parsons CL, Forrest J, Kaufman D, Evans R, Chen A, Xiao X (2008). Improvement in sexual functioning in patients with interstitial cystitis/painful bladder syndrome. J Sexu Med.

[37] Drossman DA, Patrick DL, Mitchell CM, Zagami EA, Appelbaum MI (1989). Health-related quality of life in inflammatory bowel disease. Dig Dis Sci.

[38] Simrén M, Axelsson J, Gillberg R, Abrahamsson H, Svedlund J, Björnsson ES (2002). Quality of life in inflammatory bowel disease in remission: the impact of IBS-like symptoms and associated psychological factors. Am J Gastroenterol.

[39] Mekhjian HS, Switz DM, Melnyk CS, Rankin GB, Brooks RK (1979). Clinical features and natural history of Crohn’s disease. Gastroenterol.

[40] Lichtenstein GR, Hanauer SB, Sandborn WJ (2009). Management of Crohn’s disease in adults. Am J Gastroenterol.

[41] Afzal NA, Van der Zaag-Loonen HJ, Arnaud-Battandier F, Davies S, Murch S, Derkx B, Fell JM (2004). Improvement in quality of life of children with acute Crohn’s disease does not parallel mucosal healing after treatment with exclusive enteral nutrition. Aliment Pharmacol Ther.

[42] Frank L, Kleinman L, Rentz A, Ciesla G, Kim JJ, Zacker C (2002). Health-related quality of life associated with irritable bowel syndrome: comparison with other chronic diseases. Clin Ther.

[43] Van der Zaag-Loonen HJ, Grootenhuis MA, Last BF, Derkx BHF (2004). Coping strategies and quality of life of adolescents with inflammatory bowel disease. Qual Life Res.

[44] Voskuijl WP, Van Der Zaag-Loonen HJ, Ketel IJG, Grootenhuis MA, Derkx BHF, Benninga MA (2004). Health related quality of life in disorders of defecation: the Defecation Disorder List. Arch Dis Child.

[45] Sant GR, Nickel JC, Nickel JC (1999). Interstitial Cystitis and Chronic Prostatitis: the same Syndrome. Textbook of Prostatitis.

[46] Peters KM, Carrico DJ, Ibrahim IA, Diokno AC (2008). Characterization of a clinical cohort of 87 women with interstitial cystitis/painful bladder syndrome. Urology.

[47] Peters KM, Kalinowski SE, Carrico DJ, Ibrahim IA, Diokno AC (2007). Fact or fiction—is abuse prevalent in patients with interstitial cystitis? Results from a community survey and clinic population. J Urol.

[48] Goldstein HB, Safaeian P, Garrod K, Finamore PS, Kellogg-Spadt S, Whitmore KE (2008). Depression, abuse and its relationship to interstitial cystitis. Int Urogynecol J Pelvic Floor Dysfunct.

[49] Pearlin LI, Menaghan EG, Lieberman MA, Mullan JT (1981). The stress process. J Health Soc Behav.

[50] Medzhitov R (2008). Origin and physiological roles of inflammation. Nature.

[51] Goffman E (1963). Stigma: Notes on the Management of Spoiled Identity.

[52] Hall NJ, Rubin GP, Dougall A, Hungin APS, Neely J (2005). The fight for ‘health-related normality’: a qualitative study of the experiences of individuals living with established inflammatory bowel disease (IBD). J Health Psychol.

[53] Brown P, Zavestoski S, McCormick S, Mayer B, Morello-Frosch R, Gasior Altman R (2004). Embodied health movements: new approaches to social movements in health. Sociol Health Ill.

[54] Charmaz K, Albrecht GL (2000). Experiencing Chronic Illness. The Handbook of Social Studies in Health and Medicine.

[55] Thoits PA (1995). Stress, coping, and social support processes: where are we? What next?. J Health Soc Behav.

[56] Groopman JE, Prichard M (2007). How Doctors Think.

[57] Buffington CA (2004). Comorbidity of interstitial cystitis with other unexplained clinical conditions. J Urol.

[58] Geronimus AT, Hicken M, Keene D, Bound J (2006). “Weathering” and age patterns of allostatic load scores among blacks and whites in the United States. Am J Public Health.

[59] Ferraro KF, Shippee TP (2009). Aging and cumulative inequality: how does inequality get under the skin?. Gerontologist.

[60] Alonso C, Santos J (2009). Editorial: a closer look at mucosal inflammation in irritable bowel syndrome: sex-and gender-related disparities—quantity, quality, or both. Am J Gastroenterol.

[61] Nowakowski ACH, Sumerau JE: **Swell foundations: gender, fundamental social causes theory, and chronic inflammation.** *Sociol Spectrum* 2015, forthcoming.

[62] McDade TW, Lindau ST, Wroblewski K (2011). Predictors of C-reactive protein in the National Social Life, Health, and Aging Project. J Gerontol B Psychol Sci Soc Sci.

[63] Link BG, Phelan J, Bird CE, Conrad PE, Fremont AM, Timmermans S (2010). Social Conditions as Fundamental Causes of Health Inequalities. Handbook of Medical Sociology.

[64] Kiecolt-Glaser JK, Gouin JP, Hantsoo L (2010). Close relationships, inflammation, and health. Neurosci Biobehav Rev.

[65] Suzman R (2009). The National Social Life, Health, and Aging Project: an introduction. J Gerontol B Psychol Sci Soc Sci.

[66] Waite LJ, Laumann EO, Levinson W, Lindau ST, McClintock MK, O’Muircheartaigh CA, Schumm LP: **National Social Life, Health, and Aging Project (NSHAP).** *National Archive of Computerized Data on Aging* 2007, http://www.icpsr.umich.edu/icpsrweb/NACDA/studies/20541.

[67] Geronimus AT (1992). The weathering hypothesis and the relationship of maternal age to birth outcome: evidence and speculations. Ethn Dis.

[68] Geronimus AT (1996). Black/white differences in the relationship of maternal age to birthweight: a population-based test of the weathering hypothesis. Soc Sci Med.

[69] O’Rand MA (1996). The precious and the precocious: understanding cumulative disadvantage and cumulative advantage over the life course. Gerontologist.

[70] O’Rand MA (2003). Cumulative advantage theory in life course research. Ann Rev Gerontol Geriat.

[71] Dannefer D (2003). Cumulative advantage/disadvantage and the life course: cross-fertilizing age and social science theory. J Gerontol B Psychol Sci Soc Sci.

[72] Mayer KU (2009). New directions in life course research. Annu Rev Sociol.

[73] Crosnoe R, Elder GH (2004). From childhood to the later years: pathways of human development. Res Aging.

[74] Bowling A (2004). Measuring Health.

[75] Freund PE, McGuire MB, Podhurst LS (2003). Health, Illness, and the Social Body: A Critical Sociology.

[76] Link BG, Phelan J (1995). Social conditions as fundamental causes of disease. J Health Soc Behav.

[77] Marmot M, Wilkinson R (2005). Social Determinants of Health.

[78] Mays VM, Cochran SD, Barnes NW (2007). Race, race-based discrimination, and health outcomes among African Americans. Annu Rev Psychol.

[79] Diamond T (2009). Making Gray Gold: Narratives of Nursing Home Care.

[80] Yang Y (2008). Social inequalities in happiness in the United States, 1972 to 2004: an age-period-cohort analysis. Am Sociol Rev.

[81] Dehle C, Larsen D, Landers JE (2001). Social support in marriage. Am J Fam Ther.

[82] Umberson D, Montez JK (2010). Social relationships and health: a flashpoint for health policy. J Health Soc Behav.

[83] Bury M (1991). The sociology of chronic illness: a review of research and prospects. Sociol Health Ill.

[84] McDonough P, Walters V, Strohschein L (2002). Chronic stress and the social patterning of women’s health in Canada. Soc Sci Med.

[85] Goldbeck L, Zerrer S, Schmitz TG (2007). Monitoring quality of life in outpatients with cystic fibrosis: feasibility and longitudinal results. J Cyst Fibros.

